# Error climate and alienation from teachers: A longitudinal analysis in primary school

**DOI:** 10.1111/bjep.12659

**Published:** 2024-01-02

**Authors:** Gabriele Steuer, Alyssa L. Grecu, Julia Mori

**Affiliations:** ^1^ Department of Psychology University of Bielefeld Bielefeld Germany; ^2^ Center for Research on Education and School Development TU Dortmund Dortmund Germany; ^3^ Department of Research in School and Instruction, Institute of Educational Science University of Bern Bern Switzerland

**Keywords:** alienation from teachers, error climate, error culture, longitudinal study, primary education

## Abstract

**Background:**

Dealing with errors in the classroom is a crucial aspect of instructional quality and has multiple consequences for students' own dealing with errors, their learning and their achievement. The available literature on error climate indicates a paucity of research on the effects of perceived error climate on social aspects such as student–teacher relationships.

**Aims:**

The aim of this longitudinal study was to examine the relationship between error climate and alienation from teachers.

**Samples:**

We conducted a study with two measurement points in primary school (Grade 5 in 2017 and Grade 6 in 2018) and two samples (*N* = 406 students in 29 classes in Switzerland and *N* = 345 students in 39 classes in Luxembourg).

**Methods:**

For scrutinizing the effect of error climate at T1 on alienation from teachers at T2, we used hierarchical linear modelling (students nested within classrooms).

**Results:**

For both samples, the results indicated that a positive error climate at T1 predicted less alienation from teachers at T2. We also found an effect of the shared error climate on alienation from teachers.

**Conclusions:**

The findings provide empirical evidence of the importance of improving how errors are handled in the classroom to prevent students' alienation from their teachers.

## INTRODUCTION

How errors are handled in the classroom is an important aspect of instructional quality and has a variety of consequences for students' own dealing with errors, their learning and their performance (e.g. Kreutzmann et al., 2014; Steuer et al., 2013; Steuer & Dresel, 2015). Literature on error climate shows that little research has been conducted on the effects of perceived error climate on the student–teacher relationship. In classrooms with a negative error climate, students are more likely to experience fear of making mistakes and feel alienated from their teachers (Brooks & Goldstein, 2008). Alienation from teachers refers to students' estrangement from key actors responsible for student learning in school and a decrease in perceived relatedness with teachers (Hascher & Hadjar, 2018). Teachers' unsupportive behaviours, such as negative reactions to errors, may increase students' alienation from teachers and subsequently even from school in general. Unsupportive teacher behaviours may also indirectly contribute to the development of fear of failure by influencing students' self‐beliefs and motivation to do well in school (Betts & Shkolnik, 2000; Eccles et al., 1993). Positive and supportive student–teacher relationships have been shown to alleviate school alienation (Ghaith et al., 2007; Grecu et al., 2022; Hascher & Hagenauer, 2010a; Mahmoudi et al., 2018). This implies that student–teacher interactions have a strong impact on academic as well as social learning experiences (Pianta et al., 2003). So far, little is known about the association between the error climate and students' alienation from teachers in primary school.

### Errors in educational settings

Errors are an integral part of learning. As schools are places where learning occurs, errors happen a lot. Yet, the absence of errors is usually considered an indicator of successful learning (e.g. Weingardt, 2004; Yerushalmi & Polingher, 2006): the more errors students make, the less likely they are to receive good grades. Therefore, students often perceive errors as negative events, often accompanied by negative emotions (Hascher & Hagenauer, 2010b; Tulis et al., 2016). Unsurprisingly, students are less engaged in finding the reason for their error or trying to figure out the underlying misconception (Dresel et al., 2013). Negative connotations prevent mistakes from being seen as the learning opportunity they could actually be. Along with the unfavourable individual reactions to errors described above, it is crucial to take into account that errors occur within a specific social context.

### Error climate

Error climate can be defined as the perception and use of errors as an integral part of the learning process in the social context of a learning environment (Steuer et al., 2013). In classrooms with a positive error climate, errors are viewed as natural occurrences and possible starting points for expanding knowledge. Teachers respond patiently and supportively and help students overcome errors by analysing the cause of the error and discussing underlying misconceptions (Soncini et al., 2021; Steuer et al., 2013). Students usually know whether it is ‘allowed’ to make errors or whether they are in an achievement situation where errors will result in poor grades (Meyer et al., 2006). On the contrary, in classrooms with a negative error climate, errors are seen as disruptive elements that should be avoided. Some teachers try to avoid errors by creating classroom activities that are largely structured and controlled. If errors happen despite these efforts, teachers often react impatiently to errors or simply ignore them. Classmates may also express negative reactions (e.g. taunting or laughing at the person who made error). Their behaviour can also be regarded as teacher related to a certain degree (e.g. rules that tolerate such behaviour; learning from models).

The relationship between error climate and different motivational and learning outcomes has been shown in previous empirical studies. As Kreutzmann et al. (2014) demonstrated in their cross‐sectional study, error friendliness can be positively associated with motivational tendencies such as learning goal orientation, self‐efficacy, enjoyment of learning, effort and grades. Steuer et al. (2013) included helplessness, anxiety and metacognition in their model and found that a positive error climate predicted less anxiety, lower perception of helplessness and more engagement in metacognition. As error climate is clearly related to motivation, it is likely that perceived error climate decreases over time along with well‐known motivational downward trends and increasingly negative perceptions of instruction as students progress to higher grade levels (e.g. Corpus et al., 2009; Gnambs & Hanfstingl, 2016; Gottfried et al., 2001; Lazarides et al., 2021; Maulana et al., 2016; Scherrer & Preckel, 2019). Furthermore, error climate has been shown to be linked to academic achievement, specifically achievement test scores in German (as the language of instruction) and English (as a foreign language) (Käfer et al., 2019; Rimmele et al., 2005). In another study, Steuer and Dresel (2015) illustrated a significant association between error climate and math achievement. To the best of our knowledge, no empirical studies to date have analysed the effects of error climate on social factors.

### Role of teachers and classmates in error situations

Students' errors during lessons—especially in whole‐class interaction—happen in a social context. Error situations are noticeable to teachers and classmates and therefore reactions from others are common occurrences, implying that errors encompass not only personal but also social dimensions (Käfer et al., 2019). Hence, learning from errors (or not) is based on the social context of the classroom, which affects students' emotions and motivation in and after error situations (Tulis et al., 2016).

In a broader conceptualization, error climate emerges in the interactions between teachers and students as well as in interactions among students. However, teachers in particular play a crucial role in shaping the error climate in the classroom. Due to their prominent position, teacher behaviour is more salient and therefore has a greater impact than the behaviour of single students. Therefore, teachers' reactions to errors have a significant impact on how teachers are perceived by students (Tulis, 2013), both on the individual level and as a shared belief on the classroom level (Steuer et al., 2013). Referring to Fauth et al. (2014), students' shared perception of error climate can be understood as one aspect of teaching quality, which shapes the learning environment affecting students' individual development. Often, teachers are uncomfortable dealing with errors, so errors are avoided or teachers' reactions to errors are negative (e.g. they become stressed or annoyed because of the error), which may result in an error‐avoidant instructional style (Rach et al., 2013). If teachers respond in negative ways – such as with unsupportive, offending or frightening behaviour – student–teacher relationships might be impaired. Because classmates observe error‐related situations and teacher behaviour, error climate emerges not only as a result of individual experiences but also based on shared perceptions of how errors are dealt with in the classroom.

Teachers are conspicuous in error situations and are often the ones who discover the errors and react to them. Teachers' own direct reactions to errors may influence students' behaviour in terms of encouraging or deteriorating certain reactions after errors occur (Steuer et al., 2013). For example, teachers may introduce a rule in the classroom that laughing at a student who has made an error is not allowed, thereby restricting students' acceptable behaviour. Because teachers play a prominent role in error situations and serve as role models, students may attribute negative emotions in error situations to teachers. While dealing with a mistake students observe the teacher and draw conclusions to their own potential.

In multidimensional approaches to error climate, the relevance of teachers becomes even more obvious. Spychiger et al. (1998) conceptualized the error climate – which they refer to as error culture – as a multidimensional construct and stressed the importance of the teacher's role. They first derived 10 theoretical dimensions, from which 3 empirical dimensions emerged, including 1 related exclusively to teacher behaviour. A more recent conceptualization by Steuer et al. (2013) assigns five subdimensions directly to teacher attitudes or behaviour (e.g. teacher support following errors), and relates three to classmates' behaviour (e.g. absence of negative classmate behaviour), which may also be influenced by the teacher to a certain degree. Making errors during class is a personal and social event (Käfer et al., 2019). Thus, it is crucial how teachers deal with errors in order to learn from them and build or maintain positive relationships. This is especially important because dysfunctional student–teacher relationships are regarded as a risk factor for school alienation (Hascher & Hadjar, 2018).

### Alienation from teachers and error climate

Values and attitudes towards schooling are a crucial factor behind educational inequality (Becker et al., 2022; Demanet & Van Houtte, 2014; Hadjar et al., 2021; Morinaj et al., 2019; Scharf et al., 2019; Täht & Paškov, 2009). As negative attitudes towards school and learning have detrimental effects on students' behaviour in and outside school, affecting their educational achievement and life course, further research on the causes of the development of negative attitudes is needed. When students develop negative attitudes and emotions towards teachers over time, they may become alienated from their teachers. Alienation from teachers constitutes one of the crucial domains of school alienation (Hascher & Hadjar, 2018). Given that teachers function as a key resource for students' well‐being in the school environment as well as determining students' academic, social and emotional development (Hamre & Pianta, 2007; Wang & Fredericks, 2014), alienation from teachers is particularly harmful.

Alienation from teachers develops over time as a result of everyday experiences in school that relate to the learning environment and social relationships (Hadjar et al., 2021; Rovai & Wighting, 2005), as well as teachers' actions in the classroom in terms of teaching style, classroom organization and emotional support (Blazar & Kraft, 2017; Gasser et al., 2018; Hagenauer & Volet, 2014). Research has shown that a non‐supportive role of teachers in the classroom and school context, feelings of not being cared for and a lack of trust in teachers' ability to facilitate learning may contribute to alienation from teachers (Hascher & Hadjar, 2018; Ifeagwazi et al., 2015; Murdock, 1999; Murdock et al., 2000; Pyhältö et al., 2010). Conversely, positive and supportive student–teacher relationships have been shown to alleviate student alienation (Ghaith et al., 2007; Hadjar et al., 2021; Hascher & Hagenauer, 2010a). In line with stage–environment fit theory (Eccles & Roeser, 2009), the alienation process is less likely to occur in a school environment where students' experiences are aligned with their developmental needs. In this regard, a lack of support from teachers (e.g. when they make a mistake) may intensify a mismatch between the school environment and the socio‐emotional and cognitive needs of students. Previous research highlights the importance of a student‐oriented supportive teaching style, which has been found to impede students' alienation from teachers (Hadjar et al., 2021; Morinaj et al., 2023). Furthermore, students' perceptions of fairness and interactional teacher justice have been shown to predict feelings of alienation (Çağlar, 2013; Hadjar et al., 2021). The role of student–teacher relationships (Ghaith et al., 2007; Hadjar et al., 2021; Hascher & Hagenauer, 2010a), teaching style (Hadjar et al., 2021; Morinaj et al., 2023) and students' perceptions of fairness and interactional teacher justice (Çağlar, 2013; Hadjar et al., 2021) in the development of alienation from teachers and school in general is well‐documented. Previous research on teachers' mindsets indicates that students are more likely to experience a fear of making mistakes and feel alienated from their teachers when there is a negative error climate (Brooks & Goldstein, 2008). However, the link between students' perceived error climate and the development of alienation is largely unexplored. In order to fill this gap, we will focus on this relationship in relation to primary school environment.

The connection between individual or shared perceived error climate and the development of alienation from teachers can be explained as follows: Referring to Hanna (2014), an error climate that is perceived as negative can have a detrimental effect on students' emotions and attitudes towards their teachers. Teachers' unsupportive behaviours such as negative reactions to errors may worsen the classroom climate, contribute to the development of fear of failure and affect students' beliefs about themselves as well as their motivation to do well in school (Betts & Shkolnik, 2000; Eccles et al., 1993). This in turn may impair the relationship between students and teachers, as students are less likely to open up to their teachers about difficulties in understanding or other problems, while at the same time enhancing alienation from teachers, as they do not feel taken seriously or cared for.

To summarize, we know quite a bit about the consequences of positive and negative error climate in terms of motivation and learning, but there is a lack of empirical data concerning emotional and social outcomes, such as alienation from teachers. Recently, scientific knowledge on the developmental processes and consequences of alienation from teachers has increased. However, more evidence is needed on adaptive antecedents in primary school (Hascher & Hadjar, 2018) to develop preventive strategies. In this sense, error climate in primary school as an integral part of classroom practices seems especially promising.

### Present study

By focusing on students in primary school, we address an age group in which error climate has not yet been sufficiently studied (Soncini et al., 2021). We believe that it is important to analyse error climate as early as the primary school years, as the foundation for lifelong learning is laid at this stage (cf. Dignath et al., 2008). When students experience a negative teacher response to their errors, negative emotions and a decrease in motivation are likely to occur. Learning to deal with errors in a constructive way at an early age promotes subsequent learning and is beneficial for personality development as well as the further course of schooling. Furthermore, error climate has been primarily analysed using cross‐sectional data. By using a longitudinal design with two measurement points, we aim to address this gap and contribute to research on error climate in primary schools.

The purpose of this study was to examine how primary school students perceive error climate in their classrooms and how students' perception of error climate is associated with students' alienation from teachers. To the best of our knowledge, there have been no empirical studies that have addressed this issue. The following research question was at the core of our study: *What is the relationship between perceived error climate and alienation from teachers among primary school students* (*Grades 5 to 6*)? In line with the idea that error climate affects social aspects of schooling (Käfer et al., 2019; Tulis et al., 2016), we assume that perceived negative error climate at the individual level at T1 predicts higher alienation from teachers at T2 [H1]. Furthermore, we expect that a shared negative perception of the error climate at the classroom level at T1 predicts students' individual alienation from teachers at T2 [H2]. These hypotheses remain even when controlling for alienation from teachers at T1.

Using longitudinal data from primary school students in the Swiss canton of Bern and Luxembourg, we aim to contribute to comparative research and the generalizability of the results. The two education systems exhibit substantial similarities: primary school comprises 6 years, during which the classroom teacher plays a major role in everyday school life. After primary school, students are allocated into two or more different tracks of secondary schooling. Therefore, both education systems are classified as highly stratified (Backes & Hadjar, 2017; Buchmann et al., 2016; Combet, 2019). Although both systems provide for some permeability between the tracks, the flexibility for track changes in Grades 7 to 9 is greater in the Swiss Canton of Bern, compared to Luxembourg, where 94% of students remain in their track (Krolak‐Schwerdt et al., 2015).

## MATERIALS AND METHODS

### Participants and procedure

The sampling procedure targeted primary schools in urban and rural areas in the chosen countries. Within the selected classes, participation was voluntary and subject to parental approval. Participants in this study were 406 primary school students in 29 classes in Switzerland (T1: 54.4% female; *M*_age_ = 10.3 years [*SD* = 1.00]; background: 48.8% with the child and/or at least one parent born outside Switzerland) and 345 school students in 39 classes in Luxembourg (T1: 46.1% female; *M*_age_ = 10.8 years [*SD* = .71]; migration background: 74.2% with the child and/or at least one parent born outside Luxembourg), who participated in a longitudinal research project *‘*School Alienation in Switzerland and Luxembourg’ (SASAL; 2015–2019). Primary school students voluntarily completed the paper‐and‐pencil questionnaire in their classrooms during the first half of the school year in Grades 5 (2017) and 6 (2018). In both school systems, students have the same teachers for Grades 5 and 6. The SASAL project was approved by the Swiss National Science Foundation (SNSF) and the Ethics Review Panel of the University of Luxembourg.

### Measures

#### Error climate

In order to assess the perceived error climate, we adopted three items from Baumert et al. (2009), after careful validation as part of a pilot study in both educational systems in 2015. The items can be allocated within the multidimensional model of error climate by Steuer et al. (2013) (‘Our teachers are patient when someone makes a mistake in class’ [teacher support following errors], ‘For our teachers, it is nothing bad to make mistakes’ [error tolerance by the teacher] and ‘Our teachers take care that no one is laughed at for making a mistake’ [absence of negative classmate reactions]). Participants responded on a 4‐point Likert‐type scale ranging from 1 (disagree) to 4 (agree), with higher scores indicative of a more positive perception of the error climate. Cronbach's alphas for all measures and both countries can be found in Table 1.

**TABLE 1:** Descriptive Statistics and Bivariate Correlations of Study Variables in Both Countries.

	*M*	SD	*α*	ICC	(1)	(2)	(3)	(5)	(6)	(7)
Luxembourg										
(1) Teacher alienation T2	1.62	.65	.87	.12						
(2) Teacher alienation T1	1.48	.53	.81	.08	.39***					
(3) Error climate T1	3.26	.67	.67	.07	−.33***	−.44***				
(4) Aggregated error climate T1	3.26	.29	–	–	−.26***	−.25***	.41***			
Switzerland										
(5) Teacher alienation T2	1.47	.52	.89	.11						
(6) Teacher alienation T1	1.39	.45	.87	.10				.44***		
(7) Error climate T1	3.44	.64	.78	.14				−.34***	−.48***	
(8) Aggregated error climate T1	3.44	.28	–	–				−.18***	−.31***	.44***

*** *p* < .001.

#### Alienation from teachers

We assessed students' alienation from teachers with eight items from the School Alienation Scale (SALS; Hascher & Hadjar, 2018; Morinaj et al., 2017). Sample items are ‘My teachers get on my nerves’ or ‘I don't feel taken seriously by my teachers’. Students responded to the statements on a 4‐point Likert scale from 1 (disagree) to 4 (agree), with higher scores indicative of higher degrees of alienation.

#### Control variables

Control variables included student gender (1 = female, 2 = male) and migration background. Students' migration background was measured based on the country of birth of students and their parents. The items were combined into the categories 0 = no migration background (children and parents born in Switzerland or Luxembourg, respectively) and 1 = migration background (child and/or at least one parent not born in Switzerland or Luxembourg, respectively).

### Data analysis

Analyses were conducted separately for each of the two countries. For H1 and H2, longitudinal data were analysed using hierarchical linear modelling (Singer & Willett, 2003). This allows for a process‐oriented analysis of error climate and its associations with alienation from teachers in its temporal variability and for the analysis of influences of interindividual differences in the perception of error climate on alienation from teachers. A two‐level model was used in which students were clustered within classrooms. All models were estimated using MPlus 8.9 (Muthén & Muthén, 2023).

We used a bottom‐up strategy to build the models. Thus, we first estimated random‐intercepts‐only models to determine the intraclass correlation coefficients (ICCs) of the variables. Second, we included the predictor (i.e. error climate) and migration background, gender and alienation from teachers at T1 as control variables. As migration background and gender showed no significant effects, they were removed from the models. However, we retained alienation from teachers at T1 as a control variable because we empirically found that alienation from teachers at T1 is a significant predictor of alienation from teachers at T2, and it could theoretically be assumed that the construct includes stable aspects. Predictors on level 1 were inserted in group mean centred. In the final model, error climate was included on the classroom level (level 2) to determine the effects of shared perceptions in the classroom.

## RESULTS

### Descriptive statistics and ICCs

Descriptive statistics, correlations and ICCs are displayed in Table 1. The pattern of correlations was similar in both countries. As expected, alienation from teachers correlated moderately between the two measurement points. Furthermore, alienation from teachers correlated negatively with error climate, which means that a more positive perception of error climate was associated with less alienation from teachers. ICCs ranged from .07 to .14, implying that substantial variance is explained on the between level and thus multilevel analyses are required.

### Interrelations between error climate and alienation from teachers

**FIGURE 1:**
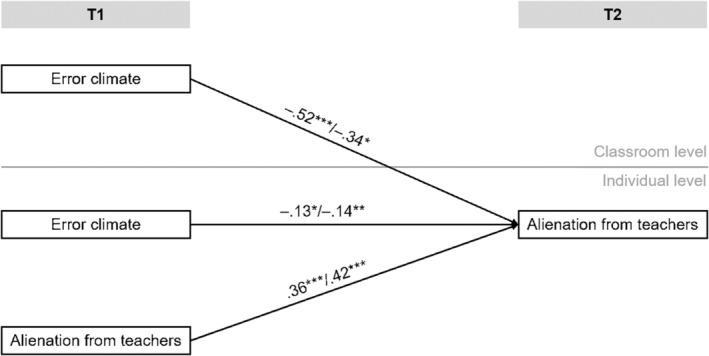
Effect of (aggregated) error climate and teacher alienation at measurement point 1 on teacher alienation at measurement point 2. Coefficients before the slash represent results from the Luxembourgish sample, coefficients after the slash refer to the sample from Switzerland. *p < .05; **p < .01; ***p < .001.

The analyses showed that error climate at T1 predicted alienation from teachers at T2 (see Figure 1). Thus, students who perceive a positive error climate from their teachers in class show less alienation from teachers 1 year later. This was true for the Luxembourgish and Swiss samples. The effects remained robust after controlling for alienation from teachers at T1. However, alienation from teachers at T1 was the major predictor of alienation from teachers at T2. The results therefore supported the first hypothesis. Results regarding the importance of the shared perceptions of error climate at the classroom level differed slightly between the two samples. The shared perception of error climate was stronger for the Luxembourgish sample, and somewhat lower, but still significant, for the Swiss sample. For both samples, it can be stated that alienation from teachers increased when the perception of error climate on the classroom level was low. The second hypothesis that the error climate on the classroom level predicts alienation from teachers at T2 was confirmed.

## DISCUSSION

The aim of this study was to provide new insights into the nature of the relationship between perceived error climate and alienation from teachers based on samples from two different countries. To our knowledge, this is the first study to investigate the association between error climate and alienation from teachers longitudinally. Furthermore, by analysing data from primary school samples, this study contributes to research on the role of teachers in the development of school alienation, which to date has mainly focused on secondary schooling.

Regarding the relationship between error climate and alienation from teachers, we found that error climate is indeed a predictor of alienation from teachers. There is a paucity of empirical evidence with a clear focus on this association. However, this finding is in line with previous research in the field of error climate. A positive error climate is associated with favourable outcomes such as more adaptive individual dealing with errors, more positive motivational tendencies, use of self‐regulated learning strategies, learning behaviour and achievement (e.g. Grassinger et al., 2018; Käfer et al., 2019; Kreutzmann et al., 2014; Soncini et al., 2022; Steuer et al., 2013). On the contrary, negative perceptions are often accompanied by dysfunctional aspects such as anxiety, feelings of helplessness and destructive learning behaviour (e.g. Grassinger & Dresel, 2017; Oser & Spychiger, 2005; Steuer et al., 2013).

Our findings on the importance of shared or individual perceptions of the error climate differ only slightly between the two country settings. It is notable that we were able to find effects at the classroom level. Often small effects of shared error climate on outcome variables have been documented, but the effect of individual error climate seems to be stronger and more consistent (e.g. Steuer et al., 2013). Compared to a classroom situation in which various classmates witness a reprimand for an error, the individual experience of a negative teacher reaction to student's error may shape the psychological reality of individual students and thus have a stronger influence on their individual development. The formation of this psychological reality needs further scientific attention in order to assess the effect of differentiated teacher behaviour, individual student characteristics and their interplay. However, the effect of the shared perception of error climate supports the relevance of social aspects of dealing with errors in the classroom.

As social aspects have rarely been considered as outcomes of error climate, our study contributes to research on error climate and adds to the growing body of research on school alienation. Our findings illustrate that errors can be seen as a significant social event in the classroom, requiring teachers to deal with the disruption of the lesson as well as the error itself. Teachers' responses can vary widely, from ignoring errors to reacting negatively to them (e.g. Schoy‐Lutz, 2005). The way teachers respond to errors can have a significant impact on student behaviour. For students, errors are often a threatening event (Laudel & Narciss, 2023) because errors may be associated with a lack of knowledge that is visible to teachers and other students (Steuer et al., 2013).

Students' attitudes towards school and learning are predicted by teaching practices – among other aspects of school – including classroom organization, teaching style and teachers' emotional support (Blazar & Kraft, 2017; Gasser et al., 2018). Teachers' constructive handling of error situations is important for maintaining a positive classroom environment. An error‐tolerant culture and effective handling of learning errors are not only crucial for individual learning processes (Rach et al., 2013) but also influence students' perceptions of teacher support (Heinze et al., 2011). When teachers support their students and provide a safe and positive classroom environment, they simultaneously contribute to high‐quality student–teacher relationships (Koca, 2016). Not surprisingly, when students perceive their teachers' error handling as supportive and friendly this leads to more positive attitudes towards teachers and less alienation from them. This is also consistent with previous research on school alienation, which has already shown the positive effect of student‐oriented supportive teaching styles in preventing students from becoming alienated from their teachers (Grecu et al., 2022; Hadjar et al., 2021).

### Strengths, limitations and future research

This study used a longitudinal design with primary school students (Grades 5 to 6) from the Swiss canton of Bern and Luxembourg, providing insights into the pattern of relationships between error climate and alienation from teachers in two different countries. We found significant effects of error climate on alienation from teachers even when including alienation from teachers at T1, which was the largest predictor of alienation from teachers at T2, explaining most of the variance. Given the stability of alienation from teachers (see Hascher & Hadjar, 2018), the finding that significant effects of error climate remain is even more striking. It highlights that error climate serves as a meaningful predictor of alienation from teachers.

This study has several limitations that may have influenced the results and should be considered in future research. First, in order to get a more holistic picture of the process leading to alienation from teachers, other possible antecedents need to be considered, such as students' motivational tendencies or peer group dynamics in the classroom. Second, by using more intensive longitudinal data with more measurement points over a longer period of time, future studies may be able to provide a deeper analysis of the underlying mechanisms behind the relationship between error climate and alienation from teachers. Third, another limitation of this study refers to the unidimensional scale used to measure error climate. This was a parsimonious approach used to obtain longitudinal data from a relatively large sample of students. Nevertheless, a broader operationalization of error climate in future research would provide more insight and would therefore be a desirable next step. Fourth, we selected only one of the three domains of school alienation, the one that theoretically best fits error climate. Future studies could include the other two domains of school alienation (i.e. alienation from classmates and alienation from learning) and their (potential) antecedents. These findings would improve our understanding of how to prevent school alienation as a whole and not just in relation to a specific domain.

### Conclusion

This study indicated a negative association between error climate and alienation from teachers. Therefore, it seems to be fruitful to promote a positive error climate already in primary school. This could be achieved through different means, such as teaching students how to deal with errors effectively or including error handling directly in the teacher training curriculum (e.g. O'Dell, 2015; Soncini et al., 2021). Furthermore, we could shed light on the relationship between error climate and alienation from teachers in primary schools, highlighting the beneficial role of a positive error climate for students' attitudes towards their teachers as well as a classroom environment conducive to learning. Future research could extend the findings of this study by exploring the reasons for changes in error climate, assessing the development of error climate over time and developing intervention programmes to promote a positive error climate in the classroom. Taken together, our study highlights the need to create a learning environment in which errors are used as learning opportunities in order to promote learning, which is also beneficial for the development of meaningful and healthy social relationships with teachers in the long term.

## AUTHOR CONTRIBUTIONS

**Gabriele Steuer:** Writing – original draft; writing – review and editing; formal analysis; software; visualization; data curation; methodology; conceptualization. **Alyssa L. Grecu:** Conceptualization; investigation; writing – original draft; writing – review and editing; data curation; project administration; methodology; software; formal analysis. **Julia Mori:** Project administration; conceptualization; investigation; methodology; writing – original draft; writing – review and editing; data curation; validation.

## FUNDING INFORMATION

This research was supported by grants from the Swiss National Science Foundation (Grant Number 100019L_159979) in Switzerland and the Luxembourg National Research Fund (Grant Number INTER/SNF/14/9857103) in Luxembourg.

## CONFLICT OF INTEREST STATEMENT

All authors declare that they have no conflicts of interest.

## Data Availability

The data that support the findings of this study are available from the corresponding author upon reasonable request.
